# Transcriptomic Landscape of Colorectal Mucinous Adenocarcinoma has Similarity with Intestinal Goblet Cell Differentiation

**DOI:** 10.2174/0113892029312303240821080358

**Published:** 2024-09-02

**Authors:** Jianbo Liu, Siyuan Qiu, Xiaorui Fu, Bin Zhou, Ruijuan Zu, Zhaoying Lv, Yuan Li, Lie Yang, Zongguang Zhou

**Affiliations:** 1 Division of Gastrointestinal Surgery, Department of General Surgery, West China Hospital, Sichuan University, Chengdu, Sichuan, 610041, China;; 2 Institute of Digestive Surgery of Sichuan University, West China Hospital, West China School of Medicine, Sichuan University, Chengdu, Sichuan, 610041, China;; 3 Colorectal Cancer Center, Department of General Surgery, West China Hospital, Sichuan University, Chengdu, Sichuan, China

**Keywords:** Colorectal cancer, mucinous adenocarcinoma, LncRNA, WGCNA, intestinal goblet cells, immune microenvironment

## Abstract

**Introduction:**

Colorectal mucinous adenocarcinoma (MC) differs from adenocarcinoma (AD) in clinical features and molecular characteristics. The current treatment of colorectal MC is not precise enough, and the molecular characteristics remain unclear. The study aims to explore the difference between colorectal MC and AD on the transcriptome level for the possibility of treating colorectal MC precisely.

**Methods:**

The data of colorectal cancer (CRC) patients from The Cancer Genome Atlas (TCGA) database was assessed, and then differential analysis and weighted gene co-expression network analysis (WGCNA) were performed to identify the differential hub RNAs between colorectal MC and AD. Differential hub lncRNAs and hub RNA of significant modules were validated by quantitative real-time PCR (qRT-PCR) among different colon cancer cell lines.

**Results:**

In total, 1680 differential expressed RNAs (DERs) were found by comparing colorectal MC (52, 13.3%) with AD (340, 86.7%). Through the WGCNA, a mucin-associated RNA module was identified, while some others might be associated with unique immune progress. Finally, 6 differential hub RNAs in the mucin-associated RNA module (*CTD-2589M5.4, RP11-234B24.2, RP11-25K19.1* and *COLCA1*) were validated by qRT-PCR and showed higher expression levels in mucin-producing colorectal cell lines (Ls174T and HT-29).

**Conclusion:**

This study suggests that clinical treatments for colorectal MC should be differentiated from AD. Further exploration of enterocyte (goblet cell) differentiation with tumor genesis and the distinct immune progression of MC may help to identify key therapeutic targets for colorectal MC. Further research on the application of immunotherapy to colorectal MC is needed.

## 1 INTRODUCTION

Colorectal cancer (CRC) ranks third and second in terms of new cases and deaths due to all cancers worldwide [1]. Colorectal mucinous adenocarcinoma (MC) is a distinct subtype of CRC, defined as abundant mucinous components comprising at least 50% of the tumor volume [2]. Although the main component is adenocarcinoma (AD), and MC accounts for only about 10% of CRC [3, 4], the clinical features of MC are more malignant than AD [2]. Compared to AD, MC occurs more frequently in the right colon, and MC patients present at a more advanced stage [5] and are more resistant to chemotherapy [6-8]. In addition, MC has a higher rate of microsatellite instability (MSI) or high-frequency microsatellite instability (MSI-H) than microsatellite stability (MSS) [8, 9]. Recent studies have revealed the distinct genomic landscape of MC, such as more mutations in *KRAS, BRAF* and *PIK3A* and higher expression of MUC families such as *MUC2* and *MUC5A*C [9, 10]. It is complicated when it comes to the survival of MC patients, with studies showing conflicting results [2]. Although studies have demonstrated the clinical and molecular differences between MC and AD, the unclear genesis mechanism of MC makes it difficult to accurately treat MC in practice. Much more work needs to be done.

To date, RNA sequencing (RNA-seq) has been used in several studies to analyze cancer [11]. The Cancer Genome Atlas (TCGA) is a public database that is well known for the availability of RNA-seq data for most cancers and provides analyzable mRNA and long non-coding RNA (lncRNA) data. Weighted gene co-expression network analysis (WGCNA) is an effective data mining method for gene screening by clustering the genes with similar expression patterns, summarizing the modules with intramodular hub genes, and relating the modules to specific clinical features [12]. Importantly, WGCNA provides an effective way to explore genes with correlation and screen genes correlated with specific clinical traits [13]. To the best of our knowledge, studies exploring mRNA and lncRNA modules of colorectal MC have not been reported.

LncRNA is a class of transcripts longer than 200 nucleotides that cannot be translated into proteins [14]. With the function of interacting with DNA, mRNA and proteins, lncRNAs can regulate gene expression at pre-transcriptional, transcriptional and post-transcriptional levels [15]. Deregulation of lncRNAs is associated with every cancer [16]. In CRC, several lncRNAs have been proven to affect cell characteristics such as invasion, apoptosis, and autophagy [17]. However, to our knowledge, lncRNA-related studies on colorectal MC are scarce, and considerable work is still required.

The aim of this study is to find the difference between colorectal MC and AD. The differentially expressed RNAs (DERs) were identified in each cancer. WGCNA network was constructed, and module-trait relationships were explored for the significant modules associated with MC. The differential hub RNAs were further identified based on the significant modules to find the potential key mRNAs and long non-coding RNAs (lncRNAs) leading to this difference. Many other analyses were performed to explain this further. Finally, quantitative real-time PCR (qRT-PCR) was used to validate the selected RNA expression level in different cell lines. Thus, these analyses may provide an explanation for the different RNA expression patterns of colorectal MC *versus* AD.

## 2 MATERIALS AND METHODS

### 2.1 Study Design and Data Processing

The study included RNA-seq counts data accessing and aggregating from TCGA database v29.0 and bioinformatics analysis such as differential analysis and WGCNA.

Based on the clinic file, the RNA-seq counts data whose primary diagnosis displayed “Adenocarcinoma, NOS” and “Mucinous adenocarcinoma” from program TCGA-COAD/ READ was obtained from the TCGA database. All the data were annotated by the official sample quality annotation file (https://gdc.cancer.gov/about-data/publications/pancanatlas) and samples were excluded in the following cases: 1) ‘TRUE’ structure appeared in column ‘Do_not_use’; 2) prior or synchronous other malignancy (for whether the tumor is metastasis, recurrence or primary is unclear), neoadjuvant therapy received, or molecular analysis/pathology is outside specification can be known from column ‘patient_annotation’. Finally, according to the tumor location displays in column ‘site_of_resection_or_biopsy’, data appeared as splenic flexure of the colon, descending colon, sigmoid colon, rectosigmoid junction, and rectum, and NOS were grouped into left-side colon and cecum, ascending colon, and hepatic flexure of the colon were grouped into the right-side colon. In consideration of the National Comprehensive Cancer Network (NCCN) guidance [18] and the difficulty of classification of data on the colon, NOS and transverse colon were excluded from the study. The microsatellite information was obtained from the online database Firebrowse (http://firebrowse.org) and attached to the TCGA clinic file. All the samples with unclear clinical information were excluded from this study.

Because the data were obtained from the TCGA database, no ethical issues were involved in the study.

### 2.2 Identification of DERs

The DERs between MC and AD were identified by the “DESeq” function in the DESeq2 package (ver. 1.32.0) of R(19), setting the log2FoldChange > 1 and raw *p*-value < 0.05. Considering of TCGA data being used, the batch effect was included in the analysis of all 60483 RNAs. According to the gene information file (https://gdc.cancer.gov/about-data/gdc-data-processing/gdc-reference-files; https://m.ensembl.org/info/genome/genebuild/biotypes.html), the DEGs were annotated with their names and types. The following nine types were regarded as lncRNA: 3prime_overlapping_ncrna, antisense, bidirectional_promoter_lncRNA, lincRNA, macro_lncRNA, non_coding, processed_transcript, sense_intronic, sense_overlapping.

### 2.3 Construction of WGCNA

In this study, the WGCNA package (ver. 1.70-3) of R was used to construct the co-expression modules [12]. The RNAs with the top 15000 median absolute deviation (MAD) were first selected. The adjacency matrix was computed as follows:


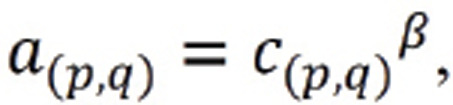


Where *α*_(*p,q*)_ represents adjacency between gene p and gene q, *c*_(*p,q*)_ represents Pearson’s correlation between gene p and gene q, and β represents the soft threshold. Next, the topological overlap matrix (TOM), representing the overlap in shared neighbors, was derived from the adjacency matrix, and the value (1 - TOM) was designated to the distance for the identification of hierarchical clustering nodes and modules. From the dendrogram, clusters were obtained by dynamic tree cutting. Finally, modules with dissimilitude < 0.25 were merged.

### 2.4 Identification of Significant Modules Associated with MC

Module eigengenes (MEs) were regarded as the major component in the principal component analysis for each module to assess the potential correlation of modules with clinical traits in the WGCNA algorithm [19]. The ‘primary diagnosis’ column in the clinic file, which contains the information that displays the patients with MC or AD, was selected as the clinical trait. Then, module-trait relationships were calculated according to the correlation between MEs and traits by single variable logistic analysis, and *P* < 0.05 was defined to be significantly correlated.

### 2.5 Identification of Hub RNAs Associate wit M Significant Modules

Hub RNAs are highly interconnected with the nodes of the module and are of functional importance. The determination of module membership (MM) to measure the correlation between the RNA and specific modules is required for hub RNA screening. For each RNA, the MM is defined by the correlation between the RNA expression matrix and the ME of the specific module. The MM measure is highly related to intramodular connectivity. Highly connected intramodular hub RNAs tend to have high MM values to the respective module. In short, the larger the MM value of the gene, the higher the correlation between the gene and a given module. In this study, the network screening function in the WGCNA package was used to identify hub RNAs. The hub RNAs were screened with a weighted q value <0.001. Only the hub RNAs that were DERs were seen as potential RNAs taking part in the differential express patterns of MC and AD. The Venn plots of DERs, hub RNAs and RNAs in significant modules were drawn by the eulerr package (ver. 6.1.1).

### 2.6 Functional Enrichment Analysis and Visualization of Differential Hub RNAs

Gene pathway analysis was generated with the g:GOSt function of g:Profiler (https://biit.cs.ut.ee/gprofiler/gost), an online tool for function enrichment analysis and conversions of gene list [20]. Statistical significance was evaluated by g:SCS algorithm, which is more suitable for g:GOSt analysis [21], and the threshold was set to 0.05. Three Gene Ontology (GO) subsets (GO molecular function, GO:MF; GO cellular component, GO:CC; GO biological process, GO:BP), Kyoto Encyclopedia of Genes and Genomes (KEGG) and Reactome pathway database were chosen in this analysis. The statistical domain scope was set to all known genes, and electronic GO annotation was allowed in this analysis.

The visualization of the outcome of enrichment analysis was performed by Cytoscape software 3.8.0. The appearance limitation was set as nodes with False Discovery Rate (FDR)-adjusted *p*-value less than 0.001 and edges with similarity more than 0.8 between nodes.

### 2.7 Construction of RNAs Co-expression Networks

To investigate the relationship between RNAs in modules associated with MC as well as the differential hub RNAs, the networks based on modules were visualized by Cytoscape software 3.8.0.

### 2.8 Heatmaps of the Expression Level of Differential Hub RNAs Across the Human Normal Cells

To further understand the characteristics of the differential hub RNAs in each module, the data of gene expression levels in normal human cells was downloaded from the human protein atlas website (https://www.proteinatlas.org), and heatmaps showing the information of these differential hub RNAs by modules were plotted by package ComplexHeatmap (ver. 2.11.1).

### 2.9 Construction of an RNA Signature Associated with MC and Calculation of Risk Score for MC

The differential hub RNAs were used to construct an RNA signature associated with MC by the least absolute shrinkage and selection operator (LASSO) analysis. The primary diagnosis (MC or AD) was set as the dependent variate. The R package glmnet (ver. 4.1-3) was utilized to fit logistic LASSO regression [22], and ten-fold cross-validation was performed to select the penalty term λ that minimizes binomial deviance. The independent variates (differential hub RNAs) reduced by LASSO were put into the logistic regression analysis by function glm in the stats package (ver. 4.1.0) for constructing the signature. Then, an external validation dataset was obtained from the Gene Expression Omnibus (GEO) database (https://www.ncbi.nlm.nih.gov/geo). After screening the database, part of the GSE2109 was chosen for validation of the signature.

For evaluation of the model efficiency, the receiver operator characteristic (ROC) curves were plotted, and area under the curve (AUC) was calculated both in TGCA and GEO datasets.

### 2.10 Survival Analysis of Differential Hub RNAs

The survival information was obtained from ‘days_to_last_follow_up’, ‘days_to_death’ and ‘vital_status’ columns in the clinic file. If the patient had already reached the status of death, then the data in ‘days_to_death’ was considered as the survival time; otherwise, the data in ‘days_to_last_follow_up’ was used. Based on the survival information and the RNAs expression data, the best cut-off value, which might lead to the smallest *p*-value in Kaplan-Meier (KM) survival analysis of these differential hub RNAs, was calculated by function “surv_cutpoint” in the survminer package (ver. 0.4.9). Then, according to the best cut-off, the RNAs were separated into a high-expression group and a low-expression group, and the KM curves were plotted by the ggsurvplot function in survminer package.

### 2.11 Cell Lines and Cell Culture

CRC cell lines Ls174T, HT-29, SW480 and HCT116 were purchased from China Center for Type Culture Collection (Wuhan, Hubei, China). According to the present studies, Ls174T and HT-29 cell lines are regarded as mucin-producing types because of the high expression of MUC2 protein, which is also the key characteristic of MC. Therefore, these two cell lines are widely applied in colorectal MC studies [23-25]. Considering that the microsatellite status could be a confounding factor of mucinous phenotype, and the microsatellite status of Ls174T (MSI) and HT-29 (MSS) is also different, two cell lines without mucinous phenotype SW480 (MSS) and HCT116 (MSI) were chosen as the control group [26]. Ls174T was inoculated to MEM (Gibco, USA) culture solution. HT-29, SW480 and HCT116 were inoculated to DMEM (Gibco, USA) culture solution. MEM and DMEM for these cell lines were complementary with 10% fetal bovine serum (Gibco, USA) and 1% penicillin-streptomycin double antibody (Gibco, USA). All cell lines were maintained at 37 °C in a humidified atmosphere containing 5% CO2, with liquid exchanging and passaging every 3–4 days.

### 2.12 RNA Extraction and qRT-PCR Analysis of Candidate RNAs

The total RNA of all the cell lines was extracted using the RNA isolater Total RNA Extraction Reagent (Cat. R401-01, Vazyme, China) according to the manufacturer's standard protocol when the cells’ confluence reached 70%-80% in a 10 cm cell culture dish. After evaluating the quantification and qualification of total RNA samples and standard nucleic acid agarose gel electrophoresis, respectively, RNA was reversely transcribed into cDNA by HiScript II 1st Strand cDNA Synthesis Kit (Vazyme, China) in terms of the manufacturer's instruction book. Then, qRT-PCR was performed using the PerfectStart II Probe qPCR SuperMix UDG (Transgen, China), TransStart Green qPCR SuperMix UDG (Transgen, China), and LineGene 9600 Fluorescent Quantitative PCR Detection system (Bioer, China). The qRT-PCR program was set to detect a maximum of 40 cycle thresholds (CT). To normalize the data, human β-actin was used as an endogenous control to balance the bias caused by different total RNA quantities. Validation of each RNA was performed in 3 duplications. Finally, the data were analyzed by the 2^-ΔΔCT^ method [27].

### 2.13 Statistical Analysis

Statistical analyses were performed with the R software 4.2.2. QRT-PCR results were analyzed with an unpaired t-test by GraphPad Prism 7.0 (GraphPad Software, San Diego, CA, USA) software. *P* < 0.05 was considered to be significant.

## 3 RESULTS

### 3.1 Characteristics of Patients and Group

**Fig. (1):**
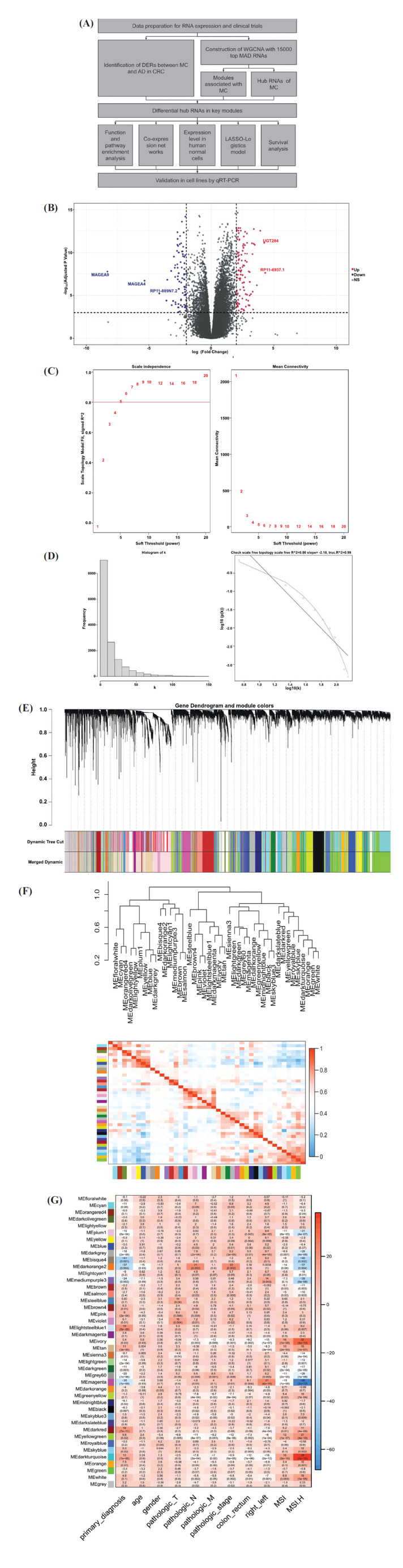
Flow chart of the study and outcome of differential analysis and WGCNA. (**A**) Flow chart of the study design. (**B**) Volcano plot of DERs, the dotted lines show the level of log_2_(FoldChange) = ± 2 and adjusted *P* value = 0.001. (**C**) The independence value and mean connectivity under different soft thresholds. (**D**) The scale-free network validation. (**E**) Hierarchical clustering tree before and after merged. (**F**) The hierarchical clustering dendrogram of the eigengenes. (**G**) The heatmap of module-trait relationships, and in each table cell the efficiency (upper) and *P* value (lower) of Logistic regression was given. It is worth noticing that the control groups of primary diagnosis, right or left and MSI-H were AD, right-side colon and MSS/MSI-L.

The study design is shown in the following diagram (Fig. **1A**). In total, data from 392 patients from TCGA was included in this study, with 52 (13.3%) MC and 340 (86.7%) AD. The detailed characteristics of the patients in this study are shown in Table **1**.

### 3.2 Identification of DERs

There were 1680 DERs between MC and AD. Five hundred and four (30.0%) DERs were downregulated, and 1176 (70.0%) DERs were upregulated. The volcano plots of both groups are shown in Fig. (**1**).

### 3.3 Construction of WGCNA

Top 15000 MAD RNAs were included in the construction of the WGCNA network. The soft threshold was determined by scale independence and mean connectivity analysis of modules with different power values ranging from 1 to 20. When the soft threshold value (β) was set to 6 in both groups, two scales had a higher independence value (R^2^) of 0.8 and lower mean connectivity and passed the scale-free network validation, as shown in Fig. (**1**) (R^2^ = 0.86, slope = -2.18). Thus, in this study, β = 6 was selected to produce a hierarchical clustering tree. After putting the screened RNAs with similar expression patterns into modules by average linkage clustering and merging the similar modules (Fig. **1**), 43 modules were identified and displayed with different colors.

### 3.4 Identification of Significant Modules Associated with MC

The eigengene adjacency network and hierarchical clustering dendrogram of the eigengenes and the heatmap of module-trait relationships are shown in Fig. (**1**). When calculating the module-trait relationship by single variate logistic, all the dependent variates were changed into binary variates as the primary diagnosis (MC *vs* AD), age (<60 *vs* ≥60), gender (female *vs* male), pathologic T (T0/Tis and T1 *vs* T2 and T3), pathologic N (N0 *vs* N1 and N2), pathologic M (M0 *vs* M1), pathologic stage (stage I and II *vs* stage III and IV), colon_rectum (colon *vs* rectum), right_left (right-side colon *vs* left-side colon), MSI (MSS *vs* MSI), and MSI-H (MSS and MSI-L *vs* MSI-H).

Finally, 23 modules with *P* < 0.05 were identified by WGCNA. According to the efficiency and *p*-value, 7 modules (darkred, lightsteelblue1, magenta, tan, darkturquoise, darkgrey and grey60) were first chosen. The darkred, lightsteelblue1, tan, and darkturquoise modules were positively correlated with MC, while the magenta, darkgrey, and grey60 modules were negatively correlated with MC.

From our preliminary findings, among these 7 modules, only darkred and lightsteelblue1 modules seemed more correlated with MC, while the other 5 modules showed higher correlation with right or left and MSI-H compared with MC. Especially, the magenta module showed a strong correlation with both the other two traits.

### 3.5 Identification of Hub RNAs Associated wit M Significant Modules

After screening the RNAs in these 6 modules by hub analysis weighted q value < 0.001 and DERs, there were 75 (darkred), 38 (lightsteelblue1), 27 (magenta), 42 (tan), 3 (darkturquoise), 2 (grey60) and 0 (darkgrey) RNAs left. As for only a few differential hub RNAs identified in darkturquoise, grey60, and darkgrey modules, they were excluded from subsequent analysis. Venn plots reflect the RNAs of common and particular based on the DERs, hub RNAs for MC, and significant modules, as shown in Fig. (**2A-E**). The differential hub RNAs in these modules share almost the same direction of regulation with the module-trait correlation.

For better interpretability, this study only focuses on the mRNAs and lncRNAs in the differential hub RNAs. Finally, 176 differential hub RNAs (73 in darkred, 37 in lightsteelblue1, 25 in magenta and 41 in tan) were taken for subsequent analysis. The list of differential hub RNAs is given in Tables **S1** and **2**.

### 3.6 Functiona Enrichmen Analysi o Significan Modules

To explore the potential reason for the difference between MC and AD, the differential hub RNAs were used for function and pathway enrichment analysis by modules. Considering some of the differential hub RNAs were not well annotated, all known RNAs and electronic GO terms were allowed in the analysis of g:Profiler.

In the darkred module, a few particular terms were enriched, while only extracellular region and extracellular space from GO:CC seemed valuable. In the lightsteelblue1 module, the terms of value such as immune system process from GO:BP, cytokine-cytokine receptor interaction from KEGG, and immune system from Reactome indicated that the lightsteelblue1 module may be an immune-associated module. Similarly, in the tan module, the immune system process from GO:BP and immune system, and the innate immune system from Reactome indicated that the tan module is also an immune-associated module. However, the differential hub RNAs in the magenta module were not able to be enriched. The enrichment result was visualized as an enrichment map network according to the relationship between terms and is shown in Fig. (**2F-H**). The appearance was limited by the nodes of FDR-adjusted *p*-value less than 0.05 and edges with similarity more than 0.8 between nodes. The size of the nodes was according to the counts of differential hub RNAs enriched into the terms, and the color of the nodes was according to the adjusted *p*-value. Nodes with the smallest *p*-value showed green, while the biggest showed purple color. The root terms of each subset were not displayed. Pathways enriched in darkred module with value are those associated with “extracellular”. In lightsteelblue1 and tan modules, pathways associated with the immune process were more frequently enriched.

### 3.7 Construction of Differential Hub RNAs Associated with MC Co-expression Networks

**Fig. (3):**
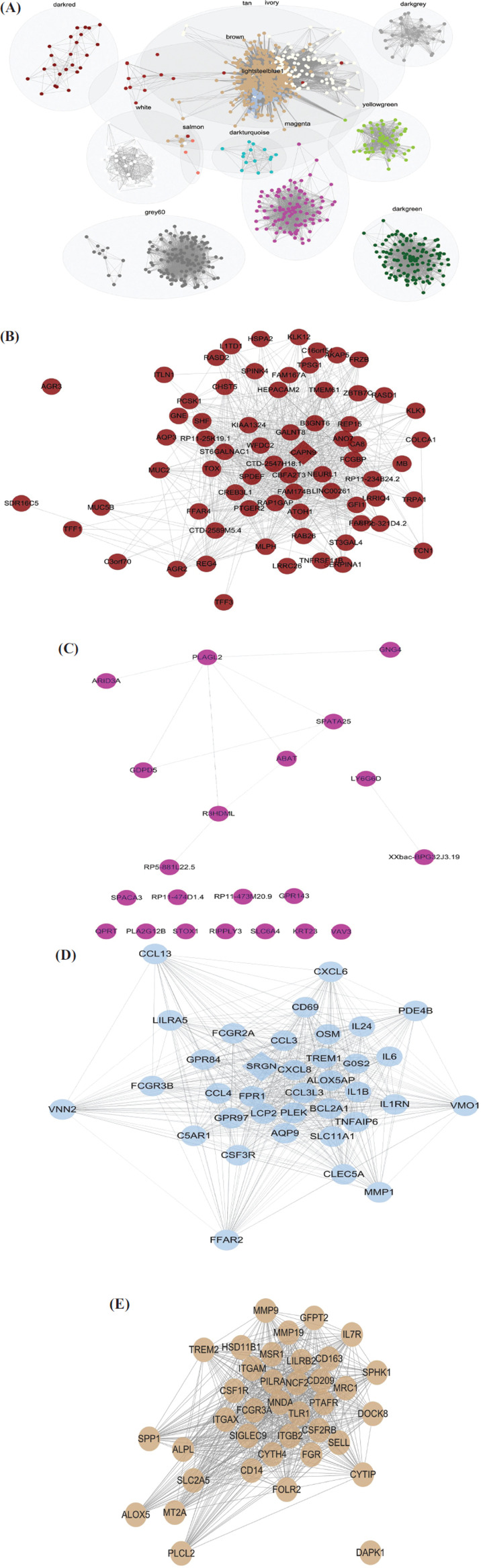
Visualization of co-expression networks. (**A**) Co-expression networks of modules associated with MC and RNAs in them. Nodes with less than the degree of 5 were hidden. (**B-E**) Co-expression networks of the four important modules with only differential hub RNAs displayed. Rhombus nodes stand by the hub RNA of the module.

All the RNAs in the 23 modules associated with MC were first included in the construction of a co-expression network. The nodes (RNAs) were grouped by modules, and the modules' co-expression network, as well as the whole RNAs network, as shown in Fig. (**3**). In the network of whole RNAs (Fig. **3A**), it can be seen that RNAs in tan module and lightsteelblue1 module connected tightly, which means that these two modules were of similar expression patterns. This corresponds with the outcome of enrichment analysis, which shows that they are all associated with the immune process. The co-expression networks of each module were also constructed, and only differential hub RNAs were displayed in the plots (Fig. **3B-E**).

### 3.8 Heatmaps of the Expression Level of Differential Hub RNAs Across the Human Normal Cells

**Fig. (4):**
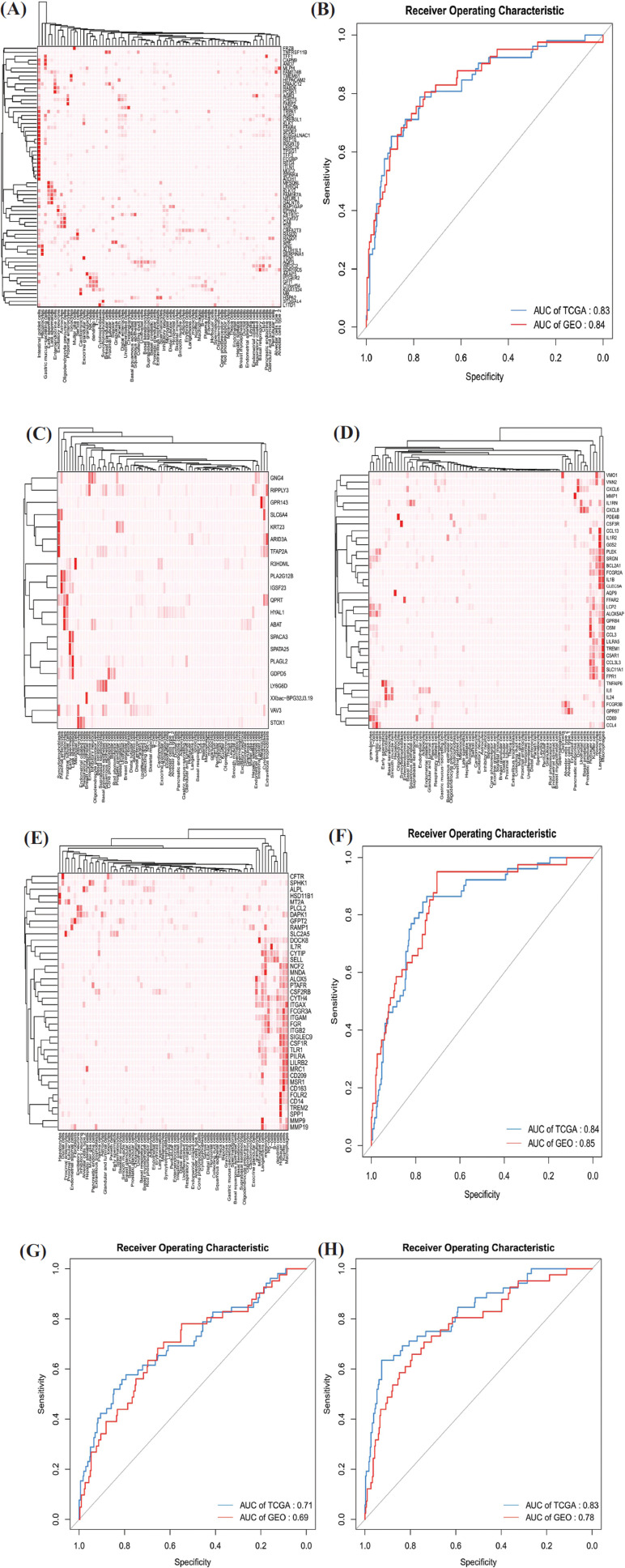
Heatmap of differential hub RNA expression level in human normal cells and ROC curves of TCGA and GEO datasets. Heatmap of differential hub RNA expression level in normal human cells of darkred (**A**), magenta (**C**), lightsteelblue1 (**D**) and tan (**E**) modules. ROC curves with AUC in the training dataset (TCGA) and validation dataset (GEO) in darkred (**B**), magenta (**F**), lightsteelblue1 (**G**) and tan (**H**) modules.

From the differential hub RNAs in each module and the former analysis, the darkred module containing most differential hub RNAs, which is reported to be associated with MC, seems to be the key module that differentiates MC from AD. While the tan and lightsteelblue1 modules are associated with the immune process, which reminds us that RNAs in these two modules may not be from the MC tumor cells but the immune cells gathered around the MC tumor cells in the MC tissues for some reason. Then, heatmaps of the expression level of the differential hub RNAs in normal human cells were plotted to verify the findings (Fig. **4A, C-E**). The outcomes showed that most differential hub RNAs in the darkred module were highly expressed in human intestinal goblet cells, while most of them in lightsteelblue1 and tan modules were highly expressed in human immune cells such as macrophages. The outcome in the magenta module also showed no specialty.

### 3.9 RNA Signature Associated with MC and Nomogram of Clinical Traits and Risk Score

LASSO analysis was performed for reducing the variates (differential hub RNAs) and the ROC curves with their AUC based on the logistic regression model of the reduced variates from four modules in TCGA and GEO datasets, which are plotted in Fig. (**4B, F-H**). The darkred and magenta modules showed the acceptable diagnostic ability of MC (AUC in darkred module: TCGA 0.83 *vs* GEO 0.84; AUC in magenta module: TCGA 0.84 *vs* GEO 0.85). The AUC of lightsteelblue1 and tan modules cannot reach 0.8 in the training and validation datasets. From this aspect, the differential hub RNAs in darkred and magenta modules seemed to have a higher value in discriminating MC from AD. More details of the logistic regression models of darkred and magenta modules are given in Table **S3**.

Considering the above-mentioned outcomes in our study, the darkred module should be the key module that differentiates MC from AD with potential clinical diagnostic value.

### 3.10 Survival Analysis

In the previous tentative analysis, this study failed to construct the LASSO-Cox regression model for the differential hub RNAs in these modules because no variates could be obtained after LASSO. This is the reason why it was chosen to calculate the best cut-off for survival of every single differential hub RNA and explain the survival outcome.

The best cut-off of 50 (darkred), 18 (lightsteelblue1), 12 (magenta) and 17 (tan) RNAs were successfully calculated in each module with significant survival difference (*P* < 0.05). Almost all the 97 RNAs showed better survival in high-expression group except for *SHF* (darkred, *P* = 0.027), *G0S2* (lightsteelblue1, *P* = 0.042), *QPRT* (magenta, *P* = 0.020), *TFAP2A* (magenta, *P* = 0.008), *CD14* (tan, *P* = 0.049), *DAPK1* (tan, *P* = 0.030), *RAMP1* (tan, *P* < 0.001), *SPP1* (tan, *P* = 0.010) and *TREM2* (tan, *P* =0.031).

However, RNAs in the darkred module, which is associated with mucin, mostly showed a positive correlation with getter survival. The KM curves of important RNAs are given in Fig. (**5**).

### 3.11 Validation of Candidate RNAs by qRT-PCR

The hub mRNA of dark red module CAPN9 and 7 differential hub lncRNAs (*CTD-2547H18.1, CTD-2589M5.4, RP11-234B24.2, LA16c-321D4.2, LINC00261, RP11-25K19.1* and *COLCA1*) were chosen for qRT-PCR validation. For each RNA, the expression of Ls174T was regarded as level 1, and comparisons were performed between Ls174 and non-mucin phenotype cell lines as well as between HT-29 and non-mucin phenotype cell lines.

**Fig. (6):**
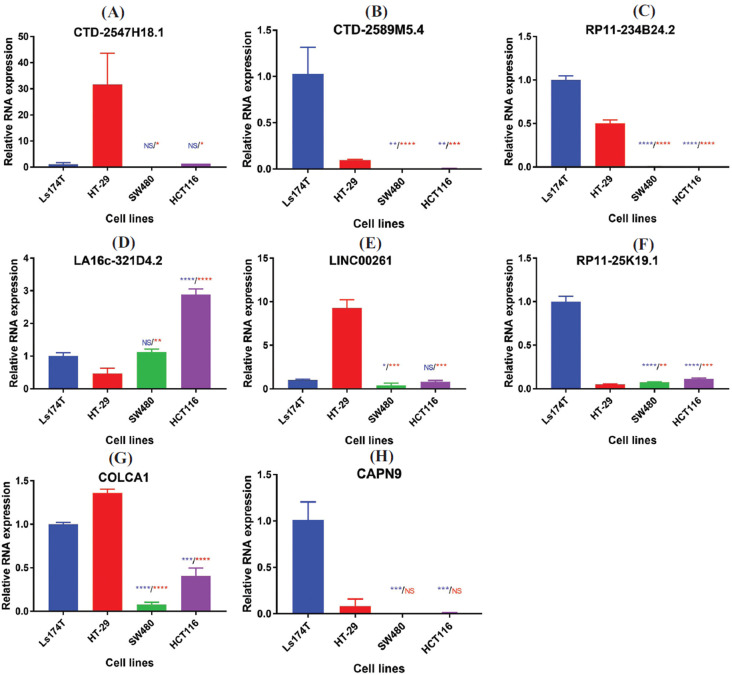
QRT-PCR validation of 7 lncRNAs and *CAPN9* in darkred module. (**A-H**) The qRT-PCR outcome of 7 differential hub lncRNAs (*CTD-2547H18.1, CTD-2589M5.4, RP11-234B24.2, LA16c-321D4.2, LINC00261, RP11-25K19.1* and *COLCA1*) and mRNA *CAPN9* *P<0.05, **P<0.01, ***P<0.001, ****P<0.0001, NS-No significance. The statistical significance outcomes are shown above the bars of SW480 and HCT116. The blue color shows the statistical significance outcomes compared with Ls174T while the red color shows that of HT-29.

Except for *LA16c-321D4.2*, all other RNAs showed higher expression in at least one mucin-producing colorectal cell line, while *CTD-2589M5.4, RP11-234B24.2, RP11-25K19.1* and *COLCA1* showed significantly higher expression level in both Ls174T and HT29 (Fig. **6**).

The primer and probe sequences applied in qRT-PCR are presented in Table **S4**.

## 4 DISCUSSION

The difference between colorectal MC and AD has already been recognized at clinical and molecular levels. However, existing studies cannot make the treatment for MC precise, and MC is still treated almost identically to AD [2]. As far as we know, the most prominent feature of MC that distinguishes it from AD is the presence of abundant extracellular mucin. Considering the malignant clinical features such as drug resistance and more frequent metastasis in MC patients, the mucins may play an important role [28, 29], but few studies have figured out the mechanism of genome/transcriptome leading to the clinical features. Meanwhile, the genesis mechanism of MC is still not clear. For more precise treatment, much needs to be explored at the molecular level of MC. This study attempts to explore the characteristics and different expression patterns of AD at the transcriptome level of MC, be the paving stone for identifying the genesis of MC, and even make MC treatment precise in the future.

In this study on constructing WGCNA and module-trait correlation, it was observed that modules have similar directions and significance of correlation with MC, right-side colon and MSI-H. According to present studies, MC is more frequent in the right-side colon and has more MSI-H than AD. Also, studies have reported that MSI-H more frequently occurs in the right-side colon [30, 31]. In the study, the relationship between MC, right-side colon and MSI-H also seems complicated, especially between MC and MSI-H at the transcriptome level. It is difficult to find the key module(s) of MC. For example, the tan, darkgrey and grey60 modules showed a coefficient and a *p*-value of high similarity in MC and MSI-H. Although from the RNAs in each module, it can be said that the darkred module is the most likely key module of MC because most of the known RNAs that are differentially expressed between MC and AD are clustered in it.

From the Venn plots (Fig. **3**), it appears that only a small fraction of the differential RNAs in the magenta and brown modules are differentially expressed between MC and AD. Combining the results of the module-trait correlations, it is consistent that the tan module does not show a better correlation with MC than MSI-H, while magenta shows a much better correlation with MSI-H. From this aspect, it can be hypothesized that the magenta module could be a potential key module of MSI-H but not MC. Although several studies have mentioned the relationship between MC and MSI-H at the molecular level, the core reason for this appearance remains unclear [32, 33]. Further studies focusing on RNAs in other modules and the mechanism of MC and MSI-H highly correlated should be conducted.

In the enrichment analysis, despite the common terms enriched in all four modules, the most specifically enriched terms of the darkred module are those with 'extracellular', such as extracellular region and extracellular space. This finding may be explained by the different hub RNAs associated with the extracellular mucins, similar to other studies [34]. The lightsteelblue1 and tan modules are both enriched for immune-associated terms, and the differential RNAs in them are mostly immune-associated RNAs. This may indicate that these two modules are both involved in the immune process. Also, from the co-expression network, the RNAs in these two modules are closely connected, which means that lightsteelblue1 and tan modules may have a similar function pattern.

**Fig. (7):**
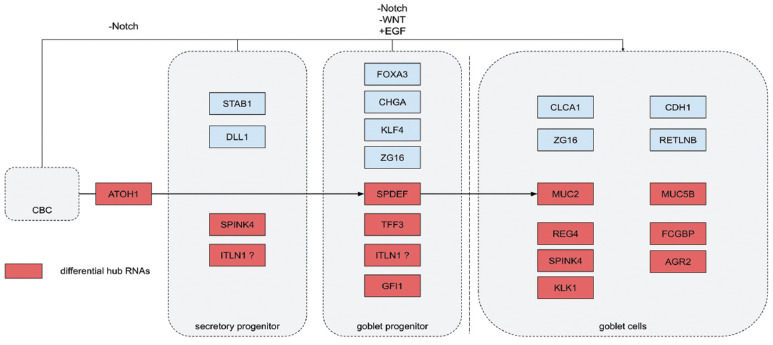
The fate of goblet cells differentiation from crypt-base columnar cells (CBC) and the marker genes in different periods*. *Because ITLN1 was reported as a marker of early goblet cells, but the proper period is not clear.

To further understand the result, the expression level of these differential hub RNAs was examined in normal human cells, and the result presents us with valuable points. Most of the differential hub RNAs in the darkred module were highly expressed in human intestinal goblet cells. It is well known that goblet cells are the major producers of mucins in the intestinal tract, and *MUC2*, the most famous mucin of MC, is secreted by goblet cells [35]. Besides, the differential hub RNAs of the darkred module also contain many of the marker RNAs of the progenitor cells of intestinal goblet cells. Points from recent studies on intestinal goblet cell differentiation have been summarized, and it was found that parts of the darkred module differential hub RNAs appear in this process (Fig. **7**) [36-39]. *ATOH1* and *SPDEF*, which are secretory lineage markers, together with other secretory and goblet progenitor markers such as *ITLN1*, *TFF3* and *GFI1* are all upregulated in our study. The mucin RNAs other than *MUC2*, such as *AGR2*, *FCGBP* and *SPINK4*, which mark intestinal goblet cells, also show this pattern. From this finding, it can be hypothesized that MC tumor cells share similar transcriptome features with intestinal goblet cells and that the genesis of MC may be associated with a specific differentiation process to goblet cells other than other types of enterocytes. Also, in this study, it was found that mRNA *FOXA2* and its neighbor lncRNA *LINC00261*, which is proven to induce *FOXA2* expression epigenetically, are also differential hub RNAs in the darkred module. *FOXA2* is proven to control the differentiation of goblet cells in mice [40, 41]. This is also evidence of the support that the genesis of colorectal MC may be related to the goblet cells’ direction of differentiation.

In the tan and lightsteelblue1 modules, the differential hub RNAs showed high levels of expression in normal human macrophages. Although these RNAs were differentially expressed between MC and AD in this study, it is more likely that these RNAs are not from tumor cells but from immune cells like macrophages. To our knowledge, there is no evidence that MC or mucins have such an effect as recruitment on macrophages that could explain the results in the study. However, there are studies that have found that MUC2 is associated with inflammation through *IL-10*, *IL-6*, and *TNF-α*. In our study, *IL-6* is one of the differential hub RNAs in lightsteelblue1. In other studies, CRC patients showed an increasing trend of *IL-6*, and the silencing of *MUC2* may increase the secretion of *IL-6* by CRC cells [42, 43]. These conclusions cannot explain the result because it was found that both *IL-6* and *MUC2* were upregulated in MC compared to AD. As a conclusion of this finding, MC is somehow more closely associated with the immune process, and macrophages may play an important role in MC. However, whether the result is caused by macrophage recruitment around tumor cells or not and whether it occurs at the transcriptome level (mucin RNAs) or protein level (mucins) should be further investigated.

The clinical value of these four modules was also investigated using the LASSO logistic regression analysis. The darkred and magenta modules show an acceptable AUC of the model. However, most of the RNAs in the models were not reported to be associated with MC, except for *TFF3*, *SPINK4* and *REG4* in the darkred module. The significance of each RNA in the TCGA and GEO datasets does not match. This result makes the clinical diagnostic value of the RNA signature evaluated in this study not sufficient for practice, but it indicates that the potential ability of the tan and lightsteelblue1 module to discriminate MC from AD is weak.

As for the survival analysis in this study, because the LASSO-Cox model could not be constructed for each module, the KM curves were plotted using the best cut-off calculated for each differential hub RNA. The result is that almost all RNAs have a better survival in the high expression group. Based on the fact that the darkred, tan and lightsteelblue1 modules are positive with MC, the result indicates that MC should have a trend of better survival for these RNAs that are upregulated in MC. This finding may explain the conflicting survival difference between MC and AD [44-46]. The malignant clinical features of drug resistance, advanced stage at diagnosis, and more metastases can be a passive strength for patient survival. At the same time, the upregulation of survival-associated RNAs found in this study shares a similar appearance with normal tissues and could be a positive strength. For example, *MUC2* is decreased in AD but increased in MC [47]. The *MUC2* in normal tissues is prominent in anti-inflammatory conditions, preventing the invasion of foreign pathogenic organisms and keeping the intestinal microecology in balance [9]. In AD, the decrease of *MUC2* may lead to a loss of protective effect and AD [48], while on the contrary, the overexpression of *MUC2* may also lead to oncogenic effects by decreasing the innate and adaptive immune response with the appearance of increasing mucin secretion [49]. The mucus layer composed of mucin may act as a physical barrier and cause resistance to systemic treatment [50]. These findings suggest that the differential hub RNAs in the darkred, light steelblue1 and tan modules may act as *MUC2*, the high expression of which makes the transcriptome or function of MC cells more similar to normal cells.

When performing qRT-PCR, the selection of cell lines was a complicated task. As far as we know, MUC2 is the marker mucin protein of the intestinal tract and is particularly expressed in goblet cells. Knowing that AD expresses less *MUC2* than normal tissue while MC expresses more, the better way to study the difference between the two subtypes is to use cell lines with high *MUC2* expression compared to those with low expression. Ls174T has been considered as a cell line with high *MUC2* expression, whereas HT-29 also expresses *MUC2* but at a moderate level [23, 51]. Because the control group is set as one MSS cell line and one MSI cell line, this study only focuses on mucinous phenotype rather than microsatellite status. From this point of view, the result of qRT-PCR suggests that lncRNAs (*CTD-2589M5.4, RP11-234B24.2, RP11-25K19.1, COLCA1*) are more highly expressed in the mucin-producing cell lines because they showed higher expression in both mucin-producing cell lines.

However, these cell lines cannot fully represent MC in the human body and may only represent part of the pathway from drivers to MUC2. This means that it will be much easier to explain the difference between mucin-producing and non-mucin-producing cell lines than the difference between Ls174T and HT-29. According to articles on the molecular differences between colorectal cell lines [26], Ls174T and HT-29 may differ in microsatellite status, CpG island methylator phenotype (CIMP) and mutations. In any case, further studies on the genesis of colorectal MC are needed to reach this conclusion.

Limitations are still presented in this study. Most of the differential hub RNAs in the four modules are not well studied, and their molecular function is not clear, especially in MC. Despite the mRNAs, many lncRNAs appeared as differential hub RNAs with validation of qRT-PCR in the study, but the roles they play in the mechanism of MC cannot be exactly explained. Although this study on bioinformatics analysis provides some insights and hypotheses, many more experiments at different levels should be conducted to explore more deeply.

## CONCLUSION

A differential expression pattern was found between MC and AD at the transcriptome level by WGCNA. LncRNAs *CTD-2589M5.4*, *RP11-234B24.2*, *RP11-25K19.1* and *COLCA1* are markers of MC validated by qRT-PCR. MC has similar mucin-producing characteristics to normal human intestinal goblet cells, and the genesis of MC may be associated with a specific differentiation pathway toward goblet cells. MC also has a close relationship with immune procession, and it was hypothesized that MC tissue contains more macrophages due to the mucin/mucus. To make the treatment of colorectal MC precise, working on novel therapeutic targets and the application of immunotherapy for colorectal MC could be of vital importance.

## AUTHORS’ CONTRIBUTIONS

Jianbo Liu, Yuan Li, Lie Yang and Zongguang Zhou did the conception and design of the study; Jianbo Liu, Siyuan Qiu, Bin Zhou, Zhaoying Lv and Ruijuan Zu did the provision of study materials; Siyuan Qiu finished the collection and assembly of data; Jianbo Liu, Siyuan Qiu, XF and Lie Yang analyzed the data and made the interpretation; Jianbo Liu and Siyuan Qiu performed the experiments; Jianbo Liu and Siyuan Qiu wrote the manuscript; Lie Yang and Zongguang Zhou make the supervision of the manuscript. All authors read and approved the final manuscript.

## Figures and Tables

**Fig. (2):**
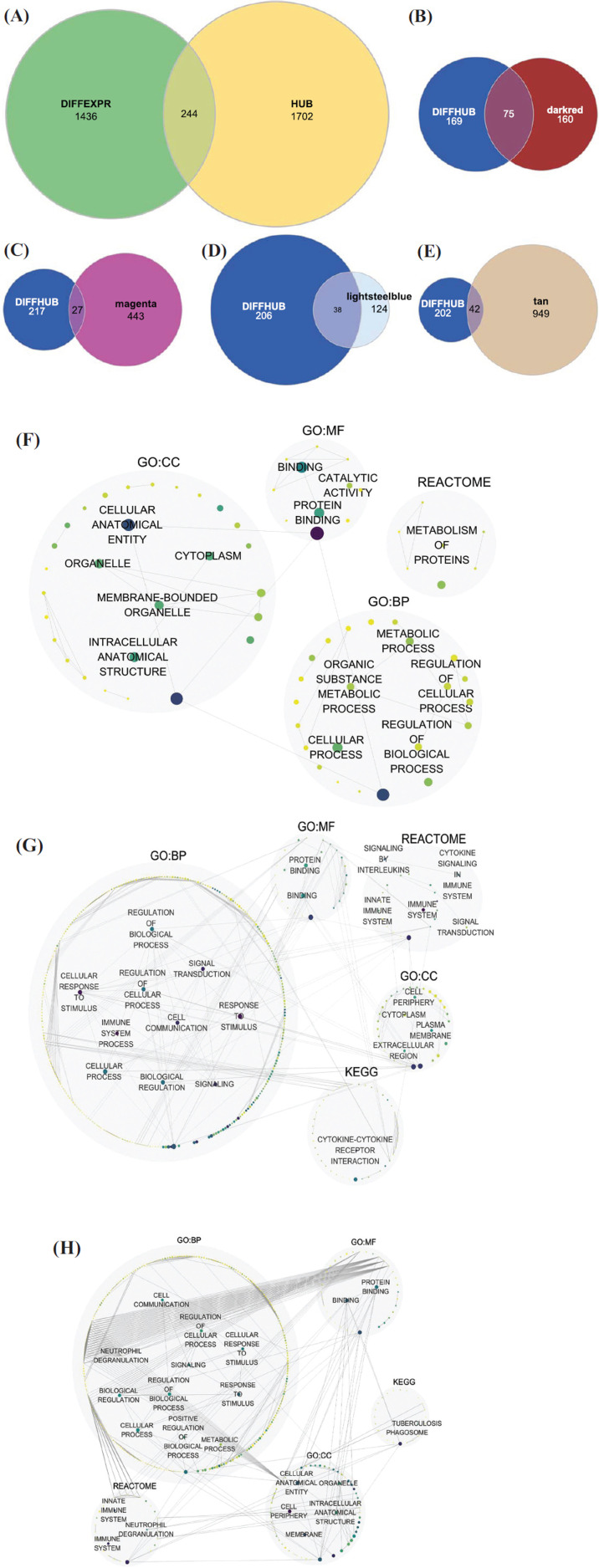
Venn plot of DERs and RNAs in significant module and visualization of the enrichment analysis. (**A**) Venn plot displaying the numbers of RNAs belonging to DERs, hub RNAs and both. (**B-E**) Venn plots displaying the numbers of RNAs belonging to differential hub RNAs, significant modules (darkred, magenta, lightsteelblue1 and tan) and both. (**F-H**) Visualization of the enrichment analysis of darkred, lightsteelblue1 and tan modules.

**Fig. (5):**
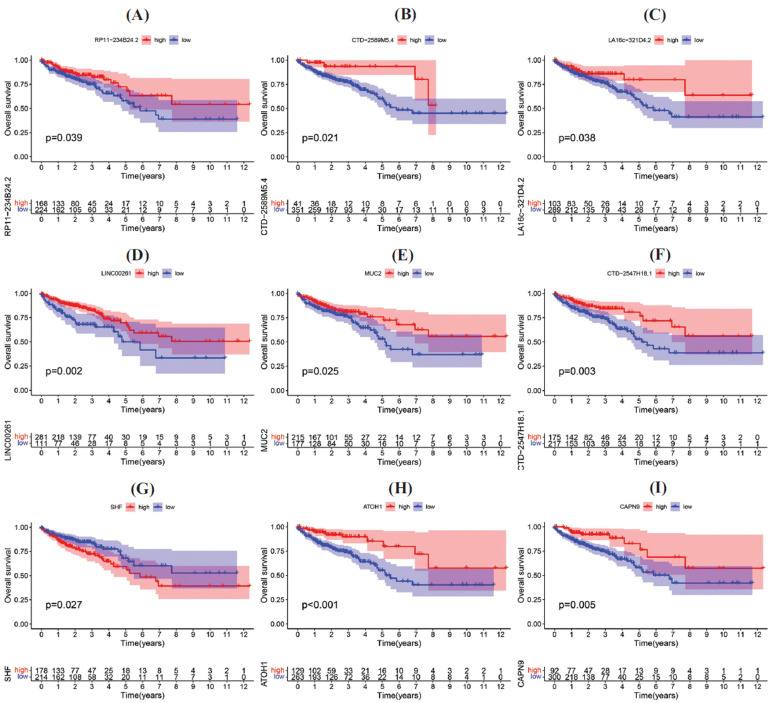
Kaplan-Meier survival curves of the part differential hub RNAs in darkred module. (**A-I**) KM survival curves of RNAs in darkred module with significant survival difference under the best cut-off where appeared the smallest *P* value.

**Table 1:** Characteristics of 392 patients and both groups.

**-**	**Level**	**Overall**	**Adenocarcinoma, NOS**	**Mucinous Adenocarcinoma**	***p***
		392	340 (86.7%)	52 (13.3%)	
Age (median [IQR])	-	67.00 [57.00,75.00]	67.00 [57.00,75.00]	66.00 [52.00,75.25]	0.477
Gender (%)	Female	190 (48.5%)	165 (48.5%)	25 (48.1%)	1
	Male	202 (51.5%)	175 (51.5%)	27 (51.9%)	-
Race (%)	American indian or alaska native	1 (0.3%)	0 (0.0%)	1 (1.9%)	0.002
	Asian	5 (1.3%)	2 (0.6%)	3 (5.8%)	-
	Black or african american	52 (13.3%)	47 (13.8%)	5 (9.6%)	-
	White	198 (50.5%)	172 (50.6%)	26 (50.0%)	-
	Not reported	136 (34.7%)	119 (35.0%)	17 (32.7%)	-
Pathologic T (%)	T0/Tis	1 (0.3%)	1 (0.3%)	0 (0.0%)	0.542
	T1	13 (3.3%)	13 (3.8%)	0 (0.0%)	-
	T2	68 (17.3%)	60 (17.6%)	8 (15.4%)	-
	T3	265 (67.6%)	229 (67.4%)	36 (69.2%)	-
	T4	45 (11.5%)	37 (10.9%)	8 (15.4%)	-
Pathologic N (%)	N0	219 (55.9%)	190 (55.9%)	29 (55.8%)	0.96
	N1	95 (24.2%)	83 (24.4%)	12 (23.1%)	-
	N2	78 (19.9%)	67 (19.7%)	11 (21.2%)	-
Pathologic M (%)	M0	332 (84.7%)	285 (83.8%)	47 (90.4%)	0.309
	M1	60 (15.3%)	55 (16.2%)	5 (9.6%)	-
Pathologic stage (%)	I	73 (18.6%)	65 (19.1%)	8 (15.4%)	0.485
	II	139 (35.5%)	119 (35.0%)	20 (38.5%)	-
	III	120 (30.6%)	101 (29.7%)	19 (36.5%)	-
	IV	60 (15.3%)	55 (16.2%)	5 (9.6%)	-
Tumor site (%)	cecum	81 (20.7%)	69 (20.3%)	12 (23.1%)	0.018
	Ascending colon	62 (15.8%)	47 (13.8%)	15 (28.8%)	-
	Hepatic flexure of colon	13 (3.3%)	10 (2.9%)	3 (5.8%)	-
	Splenic flexure of colon	5 (1.3%)	4 (1.2%)	1 (1.9%)	-
	Descending colon	13 (3.3%)	10 (2.9%)	3 (5.8%)	-
	Sigmoid colon	93 (23.7%)	89 (26.2%)	4 (7.7%)	-
	Rectosigmoid junction	64 (16.3%)	55 (16.2%)	9 (17.3%)	-
	Rectum, NOS	61 (15.6%)	56 (16.5%)	5 (9.6%)	-
MSI (%)	MSS	278 (70.9%)	245 (72.1%)	33 (63.5%)	0.007
	MSI-L	61 (15.6%)	56 (16.5%)	5 (9.6%)	-
	MSI-H	53 (13.5%)	39 (11.5%)	14 (26.9%)	-

## Data Availability

The datasets supporting the conclusions of this article are available in the TCGA (https://gdc.cancer.gov/about-data/publications/pancanatlas) and GEO (GSE103512, https://www.ncbi.nlm.nih.gov/geo).

## LIST OF ABBREVIATIONS

AD: Adenocarcinoma
AUC: Area Under the Curve
CRC: Colorectal Cancer
DER: Differential Expressed RNA
FDR: False Discovery Rate
GEO: Gene Expression Omnibus
GO: Gene Ontology
KEGG: Kyoto Encyclopedia of Genes and Genomes
KM: Kaplan-Meier
LASSO: Least Absolute Shrinkage and Selection Operator
lncRNA: Long Non-coding RNA
MAD: Median Absolute Deviation
MC: Mucinous Adenocarcinoma
MEs: Module Eigengenes
MM: Module Membership
MSI: Microsatellite Instability
MSI-H: High Frequency Microsatellite Instability
MSI-L: Low Frequency Microsatellite Instability
MSS: Microsatellite Stability
NCCN: National Comprehensive Cancer Network
qRT-PCR: Quantitative real-time PCR
RNA-Seq: RNA Sequencing
ROC: Receiver Operator Characteristic
TCGA: The Cancer Genome Atlas
TOM: Topological Overlap Matrix
WCGNA: Weighted Gene Co-expression Network Analysis
