# Supplementary material for: Transcriptomic Landscape of Colorectal Mucinous Adenocarcinoma has Similarity with Intestinal Goblet Cell Differentiation
Source: Curr Genomics. 2024 Sep 2;26(2):95–117. doi: 10.2174/0113892029312303240821080358 (PMC12105302; doi:10.2174/0113892029312303240821080358)
Supplement: Supplementary file 1 [file CG-26-2-95_SD1.pdf]

Supplementary Material

Transcriptomic Landscape of Colorectal Mucinous Adenocarcinoma has Similarity with Intestinal Goblet Cell Differentiation

Jianbo Liu[1,2,#], Siyuan Qiu[1,#], Xiaorui Fu[3], Bin Zhou[2], Ruijuan Zu[2], Zhaoying Lv[2], Yuan Li[2,*], Lie Yang[1,*] and Zongguang Zhou[1,2]

[1]*Division of Gastrointestinal Surgery, Department of General Surgery, West China Hospital, Sichuan University, Chengdu, Sichuan, China;* [2]*Institute of digestive surgery of Sichuan university, West China hospital, West China school of medicine, Sichuan university, Chengdu, Sichuan, China;* [3]*Colorectal Cancer Center, Department of General Surgery, West China Hospital, Sichuan University, Chengdu, Sichuan, China*

Table S1. Top 10 log2FoldChange differential hub mRNAs in each key (darkred, magenta, lightsteelblue1 and tan) modules.

Module	Gene_id	Gene_name	MC_hub_qweighted	MC_log2FoldChange
darkred	ENSG00000122711.7	SPINK4	0	3.40289849
darkred	ENSG00000198788.8	MUC2	0	2.916648082
darkred	ENSG00000198488.9	B3GNT6	0	2.858291268
magenta	ENSG00000244355.6	LY6G6D	2.15633E-08	-2.718530529
darkred	ENSG00000179914.4	ITLN1	1.83908E-07	2.709057253
darkred	ENSG00000134193.13	REG4	0	2.696867102
magenta	ENSG00000250641.1	XXbac-BPG32J3.19	1.21726E-06	-2.659478488
darkred	ENSG00000165272.13	AQP3	0	2.552443149
darkred	ENSG00000175426.9	PCSK1	2.49746E-05	2.495441122
darkred	ENSG00000130035.5	GALNT8	0	2.476524115
darkred	ENSG00000134827.6	TCN1	2.02433E-06	2.39818755
darkred	ENSG00000174236.3	REP15	0	2.356123278
magenta	ENSG00000138308.5	PLA2G12B	2.96085E-09	-2.152414103
magenta	ENSG00000141316.11	SPACA3	5.46972E-05	-2.145914334
lightsteelblue1	ENSG00000126262.4	FFAR2	0	2.096199388
tan	ENSG00000118785.12	SPP1	6.75227E-06	1.98509697
lightsteelblue1	ENSG00000125538.10	IL1B	0	1.957351052
lightsteelblue1	ENSG00000124731.11	TREM1	0	1.92323943
lightsteelblue1	ENSG00000103569.8	AQP9	0	1.739317959
magenta	ENSG00000168243.9	GNG4	2.97132E-12	-1.735286593
lightsteelblue1	ENSG00000258227.5	CLEC5A	0	1.716495312
lightsteelblue1	ENSG00000162747.8	FCGR3B	3.6499E-12	1.686391104
lightsteelblue1	ENSG00000099985.3	OSM	0	1.680189166
lightsteelblue1	ENSG00000171051.7	FPR1	0	1.654286955

magenta	ENSG00000101850.11	GPR143	0	-1.646341017
lightsteelblue1	ENSG00000169429.9	CXCL8	0	1.619465552
lightsteelblue1	ENSG00000136244.10	IL6	1.00591E-12	1.59704484
magenta	ENSG00000108576.8	SLC6A4	7.04109E-05	-1.569277481
magenta	ENSG00000101074.3	R3HDML	0	-1.563414665
tan	ENSG00000132329.9	RAMP1	2.43278E-06	1.54031615
tan	ENSG00000117594.8	HSD11B1	0	1.49938142
magenta	ENSG00000216588.7	IGSF23	3.77142E-07	-1.477233103
magenta	ENSG00000137203.9	TFAP2A	0	1.468919739
tan	ENSG00000131459.11	GFPT2	0	1.453905074
tan	ENSG00000177575.11	CD163	0	1.448055529
tan	ENSG00000100985.7	MMP9	1.3579E-05	1.348588047
tan	ENSG00000162551.12	ALPL	4.50997E-11	1.32264461
tan	ENSG00000107099.14	DOCK8	0	1.30406377
tan	ENSG00000169896.15	ITGAM	0	1.272604759
tan	ENSG00000154822.14	PLCL2	0	1.228766895

Table S2. Differential hub lncRNAs in each key (darkred, magenta, lightsteelblue1 and tan) modules.

Module	Gene_id	Gene_name	Gene_type	MC_hub_qweighted	MC_log2FC
darkred	ENSG00000254988.1	CTD-2547H18.1	antisense	0	2.692004116
darkred	ENSG00000259974.2	LINC00261	lincRNA	0	2.552953617
darkred	ENSG00000255007.1	CTD-2589M5.4	antisense	0	1.766259801
darkred	ENSG00000196167.8	COLCA1	antisense	1.0391E-08	1.449704444
darkred	ENSG00000262482.1	LA16c-321D4.2	antisense	1.76908E-12	1.36746028
darkred	ENSG00000255474.1	RP11-234B24.2	lincRNA	0	1.129950219
darkred	ENSG00000167912.5	RP11-25K19.1	antisense	0	1.082964173
magenta	ENSG00000146666.5	LINC00525	antisense	0.000102982	-1.137628643
magenta	ENSG00000261650.1	RP11-474D1.4	lincRNA	7.40512E-05	-1.261542491
magenta	ENSG00000226812.2	RP5-881L22.5	antisense	0	-1.296442817
tan	ENSG00000236935.1	AP003774.1	antisense	1.86746E-05	-1.723736777
magenta	ENSG00000262370.4	RP11-473M20.9	lincRNA	5.84618E-06	-2.076905179

Table S3. Logistics regression model of darkred and magenta modules in TCGA and GEO datasets.

-	Gene	TCGA			GEO		
		Coefficient	P	OR (2.50%~97.50%)	Coefficient	P	OR (2.50%~97.50%)
darkred	Intercept	-3.34745	1.76E-24	0.035174 (0.017799 ~ 0.064666)	-3.64187	4.40E-11	0.026203 (0.008433~0.074656)
	TFF3	0.000261	0.243019	1.000261 (0.999813 ~ 1.000698)	1.10E-05	0.594905	1.000011 (0.999969~1.000051)
	SPINK4	7.56E-05	0.755431	1.000076 (0.999672~1.00065)	9.48E-06	0.607855	1.000009 (0.999973~1.000047)

	REG4	0.00058	0.318479	1.00058 (0.999434~1.001745)	5.90E-05	0.021822	1.000059 (1.000009~1.000111)
	CREB3L1	0.013915	0.053666	1.014012 (0.999661~1.028511)	0.000515	0.306304	1.000515 (0.999515~1.001499)
	RP11-234B24.2	-0.0115	0.473678	0.988569 (0.957432~1.020269)	-0.00116	0.054786	0.998839 (0.997473~0.999842)
	ST3GAL4	-0.01074	0.486098	0.989321 (0.958727~1.019571)	-0.00152	0.421995	0.998477 (0.994552~1.001997)
	TNFRSF11B	0.015658	0.208238	1.015781 (0.990611~1.041078)	4.28E-08	0.999832	1 (0.999568~1.000434)
	SHF	0.037497	0.525145	1.038209 (0.922534~1.163823)	0.003831	0.032803	1.003839 (1.000314~1.007409)
	TOX	0.049456	0.3509	1.050699 (0.94307~1.168092)	0.001036	0.19331	1.001037 (0.999408~1.002554)
	MFSD6L	0.265893	0.019048	1.304596 (1.040938~1.630068)	-0.00595	0.567028	0.994073 (0.972572~1.013203)
	MB	0.072131	0.348402	1.074796 (0.918209~1.253628)	0.000527	0.463163	1.000527 (0.998865~1.001737)
	TMEM61	-0.0723	0.443222	0.930255 (0.768095~1.12316)	0.000736	0.728908	1.000736 (0.996533~1.004905)
magenta	Intercept	-0.17463	0.591206	0.839766 (0.443715~1.595682)	0.473408	0.449675	1.605457 (0.48796~5.738399)
	QPRT	-0.01394	0.335339	0.986159 (0.956723~1.012795)	-0.00054	0.243688	0.999455 (0.99846~1.000286)
	VAV3	-0.05338	0.092948	0.948023 (0.887493~1.005222)	-0.00014	0.591734	0.999861 (0.999318~1.000339)
	GNG4	-0.03737	0.184166	0.963321 (0.906232~1.013176)	-0.00365	0.058237	0.996362 (0.992177~0.999729)
	ARID3A	-0.04817	0.336099	0.952974 (0.853727~1.038412)	-0.00187	0.22557	0.998133 (0.994649~1.000605)
	PLA2G12B	-0.08241	0.260719	0.920892 (0.779624~1.045906)	-0.00032	0.897693	0.999676 (0.994209~1.004066)
	RP11-474D1.4*	0.012351	0.927351	1.012427 (0.751166~1.292874)			
	STOX1	-0.40817	0.028445	0.664863 (0.449002~0.935008)	-0.00565	0.164816	0.994371 (0.985856~1.001439)
	HYAL1	0.028381	0.170451	1.028788 (0.987112~1.072137)	0.001755	0.000172	1.001757 (1.000868~1.002731)
	R3HDML	-0.0728	0.723319	0.929788 (0.590905~1.335178)	-0.00928	0.066087	0.990765 (0.980216~0.999972)
	SPACA3	-0.47544	0.317952	0.621614 (0.172389~1.198465)	-0.00416	0.316729	0.995852 (0.987264~1.003458)
	SLC6A4	0.124568	0.700175	1.132659 (0.521105~1.927203)	0.000177	0.964184	1.000177 (0.991745~1.007509)

*Because of lacking data of RP11-474D1.4 (magenta) in GSE2109, the Logistic regression model of magenta module in GEO dataset only contained other RNAs

Table S4. Primer and probe sequences applied in qRT-PCR*

Target	Sequence (5'-3')
CTD-2547H18.1 (F)	CAGGCAGGTCATCCCAGT
CTD-2547H18.1 (R)	CAGATGGTCACTTTCTTGCT
CTD-2547H18.1 (Probe)	TCTTCTCAGGTCACTGTCCACGCAC
CTD-2589M5.4 (F)	ATGACGCACAGGAGAAAGAG
CTD-2589M5.4 (R)	TCCACCTCAGGACCCAAGA
CTD-2589M5.4 (Probe)	CTCTCTGCCTGATCCTCAGTGTCTG
RP11-234B24.2 (F)	GGGACTACAGCCTTCATTTC
RP11-234B24.2 (R)	GGTCAGTCTGAGGGATGTTT
RP11-234B24.2 (Probe)	AAAATGTTCCACGCCTTGGTCAATG
LA16c-321D4.2 (F)	CACAGGACTTAGCCTACCAT
LA16c-321D4.2 (R)	AATTCCCAGCGTTGCTTCAC
LA16c-321D4.2 (Probe)	CGGTTGTCTTTCTCCTCACAGCCTT
LINC00261 (F)	GCCGTGAAGCTAAAGGTCTC

LINC00261 (R)	TGTGGGCACAGGCATGTC
LINC00261 (Probe)	TGCCCTCTCGGTGGCTGTGG
RP11-25K19.1 (F)	AGGGCAGTGTCCTTGGTGA
RP11-25K19.1 (R)	GCTCGTATTTGCTTGGTGCT
RP11-25K19.1 (Probe)	TGGGCGACATTGTCCTCCATCTCAT
COLCA1 (F)	CAGCCGAGTTACCCGACAT
COLCA1 (R)	CTCAAACCTATACCCGAAGG
COLCA1 (Probe)	TGCAGCATCCAGCCACCGAC
CAPN9 (F)	GGTTGAGTGGAACGGGTCG
CAPN9 (R)	AGGGCATCGGGAGTGAGGT

*The qRT-PCR validation of these lncRNAs were performed with adding probe in the reaction system, while the validation of CAPN9 not.
